# Intrinsic restriction activity by apoB mRNA editing enzyme APOBEC1 against the mobility of retroelements

**DOI:** 10.1186/1742-4690-8-S2-P83

**Published:** 2011-10-03

**Authors:** Atsushi Koito, Terumasa Ikeda

**Affiliations:** 1 Department of Retrovirology and Self-Defense, Faculty of Life Sciences, Kumamoto University, Kumamoto, Japan 860-8556

## 

A large portion of the mammalian genome is derived from ancient transposable elements. Retroelements, transported by an intracellular copy-and-paste process involving an RNA intermediate (retrotransposition), constitute a majority of these mobile genetic elements. Endogenous retroviruses are LTR-type retroelements accounting for around 8% of human or murine genomic DNA. Non-LTR members are present in extremely high copy numbers; with LINE-1 contributing to nearly 40% of human and murine genomes. These LINE-1 elements modify mammalian genomes not only through insertions, but also by indirect replication of nonautonomous retrotransposons such as SINEs. As expected, cellular machineries of vertebrate’s innate immunity have evolved to support a balance between retroelement insertions that cause deleterious gene disruptions and those that confer beneficial genetic diversity. The ability of mammalian cytidine deaminases encoded by the *APOBEC3* (*A3*) *genes* to restrict a broad number of endogenous retroelements and exogenous retroviruses, including MLV and HIV-1, is now well established. The RNA editing family member apolipoprotein B (apo B)-editing catalytic subunit 1 (APOBEC1; A1) from a variety of mammalian species, a protein involved in lipid transport and which mediates C-to-U deamination of mRNA for apo B, has also been shown to modify a range of exogenous retroviruses, but it’s activity against endogenous retroelements remains unclear. Here we show that A1 family proteins can also reduce the mobility and infectivity potential of LINE-1 and LTR retrotransposons (or endogenous retroviruses) such as IAP and MusD sequences. The anti-L1 activity of A1 was mainly mediated by a deamination-independent mechanism with inhibition at step prior to the integration, and was not affected by nuclear localization of the proteins. In contrast, A1 inhibits the replication of murine IAP and MusD through a DNA deamination-dependent mechanism. Thus, the AID/APOBEC family proteins including A1s employ multiple mechanisms to regulate the mobility of autonomous retrotransposons in a wide range of mammalian species.
